# Atherosclerosis preventive effects of marrubiin against (TNF-α)-induced oxidative stress and apoptosis

**DOI:** 10.34172/jcvtr.2023.31704

**Published:** 2023-09-23

**Authors:** Ailar Nakhlband, Alireza Garjani, Nazli Saeedi, Yadollah Omidi, Samad Ghaffari, Jaleh Barar, Morteza Eskandani

**Affiliations:** ^1^Research Center of Psychiatry and Behavioral Sciences, Tabriz University of Medical Sciences, Tabriz, Iran; ^2^Research Center for Pharmaceutical Nanotechnology, Biomedicine Institute, Tabriz University of Medical Sciences, Tabriz, Iran; ^3^Faculty of Pharmacy, Tabriz University of Medical Sciences, Tabriz, Iran; ^4^Department of Pharmaceutical Sciences, Barry and Judy Silverman College of Pharmacy, Nova Southeastern University, Fort Lauderdale, FL 33328, USA; ^5^Cardiovascular Research Center, Tabriz University of Medical Sciences, Tabriz, Iran

**Keywords:** Marrubiin, Cardioprotection, Intracellular ROS, Apoptosis, Cardiovascular diseases

## Abstract

**Introduction:**

Atherosclerosis is a complicated cascade of inflammatory processes, oxidative stress, and apoptosis, making it the most prevalent cardiovascular disease. The onset and progression of cardiovascular diseases are greatly influenced by oxidative stress. Targeting oxidative stress is an effective strategy for treating such diseases. Marrubiin is a bioactive furan labdane diterpenoid acts as a strong antioxidant to protect against oxidative damage. This study aimed to investigate the protective effects of marrubiin against oxidative stress and apoptosis in a cellular model of the vascular system.

**Methods:**

Human umbilical vein endothelial cells were treated with varying concentration of marrubiin and its IC50 value was determined. The antioxidant potential of marrubiin was assessed by measuring the intracellular level of glutathione (GSH) using a colorimetric technique. Since apoptosis plays a significant role in the plaque rupture, the study also evaluated the protective effects of marrubiin on the expression of key genes involved in apoptotic pathways.

**Results:**

Cells treated with marrubiin showed increased GSH levels compared to cell therapy control cells, indicating marrubiin’s ability to counteract the effects of TNF-α’s on GSH levels. Furthermore real-time PCR analysis demonstrated that marrubiin upregulated Bcl-xl while downregulating caspase3 and Nox4 in treated cells. These findings suggest that marrubiin protects against apoptosis and oxidative stress.

**Conclusion:**

Based on our findings, marrubiin is recommended as a preventive/therapeutic treatment for diseases caused by elevated intracellular reactive oxygen species levels in cardiovascular diseases.

## Introduction

 Cardiovascular diseases (CVDs) are the leading causes of death worldwide, with their incidence continuing to rise.^1,2^ Despite advances in medical approaches, the World Health Organization (WHO) predicts that CVDs will result in over 23.6 million deaths globally by 2030.^2^ Atherosclerosis characterized by chronic inflammation of the artery wall, is a major contributor to morbidity and mortality in CVDs. Given the increasing prevalence of CVDs there is a need for earlier and more accurate identification, as well as more effective therapeutic/preventive strategies.

 Artery endothelial cells (AECs) play a crucial role in cardiovascular health. Dysfunction of AECs leads to reduced Nitric Oxide bioavailability, impaired endothelium- dependent vasodilation, increased platelet-leukocyte-endothelial cell interactions, and a cascade of events contributing to atherosclerosis.^3^ Apoptosis, programmed cell death, is known to play a significant role in the development and progression of atherosclerosis. Apoptosis not only contributes to plaque formation, but also increases the risk of plaque rupture and thrombosis. Additionally, macrophage apoptosis can lead to intraplaque thrombosis if apoptotic bodies are not scavenged. High levels of cholesterol oxides in the cell membrane activate foam cell apoptosis.^4^ Oxidized LDL-mediated apoptosis is triggered by Caspase and sphingomyelinase activation, decreased Bcl-2, and inhibition of the nuclear transcription factor-kB.^5^ Interestingly, common risk factors for atherosclerosis, such as high glucose levels, oxidative stress, and angiotensin II, induce apoptosis in AEC.

 It is well established that oxidative stress is another significant factor in the development of atherosclerosis.^6^ Although oxidative stress is required for vascular homeostasis, excessive generation of reactive oxygen species (ROS) is linked to vascular damage. ROS are key mediators of cellular injury, particularly in endothelial cell destruction. Endogenous antioxidants act as checkpoints to prevent the negative effects of ROS.^7^ Several studies have shown a close correlation between ROS production apoptosis in the plaques.^6^ TNF-α induced oxidative stress is widely known to have a significant role in CVDs. High doses of TNF-α have been shown to promote cardiomyocyte loss via necrosis or apoptosis.^8^

**Figure 1:**
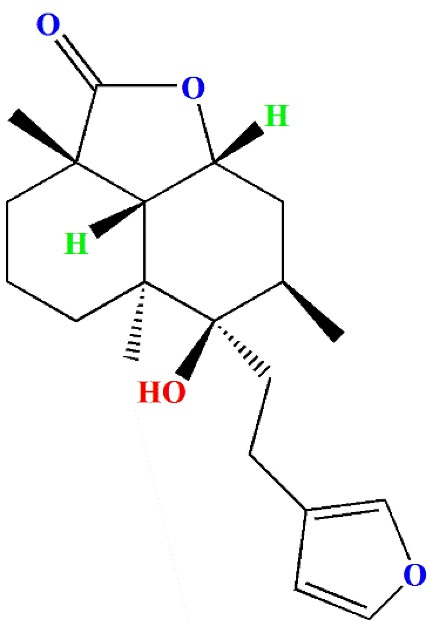
The chemical structure of marrubiin

 Antioxidants are chemicals that protect against oxidative damage even when present in extremely low concentrations. Epidemiological studies indicate that low antioxidant levels are related with an increased risk of CVD, whereas greater consumption appears to be beneficial. As a result, it is clear that nutritional supplementation and preventive medicine are successful strategies that hold tremendous potential for overcoming CVDs.^9,10^ Many traditionally significant medicinal plants contain active compounds or molecules that act as precursors to biosynthesized secondary metabolites, which may be responsible for the biological activity. One such chemical is marrubiin, a potentially useful substance found in high amounts in several traditionally important Lamiaceae species. It has shown outstanding pharmacological effects while maintaining commendably high safety margins. Marrubiin is a well-known diterpenoid lactone (Figure 1) that serves as the bitter ingredient in horehound and many other Lamiaceae medicinal herbs. It is one of the major components of *Marrubium vulgare*, *Leonotis leonurus* and *Leonotis nepetifolia*, which are used in several countries to treat various illnesses.^11^

 In our previous study, we introduced marrubiin as a potent antioxidant with cardioprotective properties that might be regarded as a viable option to combat CVDs and their health-related problems. In continuation of the previous study, in the present study, we focused more on the mechanisms of cardio protective potential of marrubiin. Therefore we assessed two important pathways in the development of atherosclerosis: apoptosis and oxidative stress. Considering the critical role of endothelial cells in atherosclerosis, human umbilical vein endothelial cells (HUVECs) were chosen as the cell line model.

## Materials and Methods

###  Materials

 HUVECs were procured from the Iranian National Cell Bank and the Pasteur Institute (Tehran, Iran). IWAKI provided cell culture flasks and plates (Chiba, Japan). Gibco (Paisley, UK) provided DMEM-F12 media, and fetal bovine serum (FBS), as well as trypsin-EDTA (0.02-0.05%). Other chemicals that were not specified above were of the finest grade and were supplied by either Sigma-Aldrich (Poole, UK) or Merck Co. (Darmstadt, Germany). SuperScript II reverse transcriptase and agarose were purchased from Gibco, Invitrogen, (Paisley, UK). Penicillin G, streptomycin, diethyl pyrocarbonate (DEPC) treated water were obtained CinnaGen, (Tehran, Iran). Ribonuclease inhibitor was obtained from Fermentas, Canada. Random hexamer primers (pdN6), Taq DNA polymerase, deoxynucleotide triphosphate monomers (dNTPs), polymerase chain reaction reaction buffer, MgCl2 and QIAquick PCR purification kit were from QIAGEN (Crawley, UK). Forward and reverse oligonucleotide primers and EGFR antisense were bought from MWG-Biotech, (Ebersberg, Germany).

###  Cell culture

 HUVECs were selected as the cell line model due to their relevance in studying endothelial cell function and their involvement in diseases such as atherosclerosis. The cells form a monolayer structure adhering to a surface. HUVECs were cultivated at 37 °C in a humidified incubator with a 95% air/5% CO2 environment. The cell culture medium was DMEM/F12 with 10% (v/v) fetal bovine serum, penicillin G (200 U/mL), and streptomycin (200 µg/mL). The cultivated cells were fed every other day and passaged when they reached 80% confluency.

 When the cells were nearing confluency, they were split every third or fourth day. During cell splitting, the old growth media was removed, and 5 mL of sterile phosphate buffered saline (PBS) was used to wash away any proteins that may impede the trypsin. PBS was then withdrawn, and the flask was incubated at 37°C for 3-5 minutes with a 1 ml 0.25% trypsin/EDTA solution. The addition of trypsin/EDTA solution causes cell detachment from the incubation flask, which may be observed using a phase-contrast microscope. After detaching the cells, growth media added to the flask to stop the effect of trypsin. Ten millilitres of cell suspension were centrifuged at 90 × g for ten minutes. The supernatant was poured out and the cell pellet was re-formed in growth medium to achieve the concentration of 1 million cells/ml. 1-2 million cellswere incubated in new ﬂask.

###  Free radical scavenging assessment

 To assess the antioxidant activity of Marrubiin, a free radical scavenging assay using 2, 2-diphenyl-1-picrylhydrazyl (DPPH) was performed. This assay helped determine the ability of marrubiin to scavenge free radicals and protect against oxidative stress. On this purpose, 1.42 mg of marrubiin analyzed by DPPH assay. Serial dilutions of marrubiin prepared (2.5, 5, 10, 20, 40, 60, 80 and 100 μg/mL) via solving in methanol. After complete mix with an equal volume of DPPH solution (0.004% w/v in absolute ethanol), the solutions were incubated for 30 min. Colour of the solutions changed due to reduction of DPPH during reaction with the antioxidant mixture. Commonly the colour change from dark violet to light yellow measured by spectroscopy. Subsequent to incubation for 30 min at a stable temperature, UV-VIS spectrophotometer applied to read the absorbance at 517 nm. Furthermore, quercetin was subjected to the same process as the positive control.

###  Glutathione status assay

 The Glutathione status of the treated HUVECs was determined using a Glutathione Assay Kit (Sigma-Aldrich). This allowed the researchers to measure the levels of glutathione, an important antioxidant molecule, in the cells after treatment with marrubiin. To this end, HUVECs were grown on 6-well plates and allowed to reach the necessary confluency. The cells were then treated for 24 hours with marrubiin (39 µM) before being stimulated with TNF-α for 24 hours. Cells were then trypsinized with 250 µL of trypsin-EDTA and centrifuged at 90 × g for 10 minutes. Cells were washed with PBS and centrifuged at 600 × g to get a packed cell pellet. Sulfosalicylic Acid % 5 (SSA) solution (3 volumes of cell pellet) added to the packed cell pellet. The suspension was frozen in liquid nitrogen and thawed twice in a 37 °C bath, and the extract was centrifuged at 10000 × g for 10 minutes. Finally, the absorbance of the samples was determined using the spectrophotometer at 412 nm.

###  RNA isolation

 mRNA expression levels were determined by extracting total RNA from the cells using Trizol reagent. This helped identify any changes in gene expression related to apoptosis and oxidative stress pathways after treatment with marrubiin.

 Total RNA was extracted to determine mRNA expression levels. To this end, the cells were seeded in 6 well culture plates and treated with marrubiin (39 M) prior to TNF-α induction. After 24 hours of TNF-α stimulation, the cells were lysed with 1 ml of the Trizol reagent.

 Chloroform was added to the cell lysate and samples were centrifuged at 12,000 g for 15 minutes at 2-8 °C. The colorless upper aqueous phase (containing RNA) was transferred to a new tube after phase separation, and 0.5 ml of 2-propanol was added to each sample. RNA precipitated throughout the 10 minutes of 12,000 g centrifugation at 2-8 °C, forming a pellet on the tube’s side and bottom. After being dried, the RNA pellet was washed twice with 1 ml of 75% ethanol before being dissolved in DEPC water.

###  Reverse Transcription Polymerase chain reaction

 The RNA samples were isolated, measured using a NanoDrop® ND-1000 spectrophotometer, and then 1 µg of each extracted RNA sample was reverse transcribed into complementary DNA (cDNA) using BioFact^TM^ 2X OneStep RT-PCR Pre-Mix. A mixture containing RNA, a primer, 2X RT Pre-Mix, and RNase-free water were made (20 µl), and the mixture was incubated for 5 min at room temperature (24 ± 2°C). The mixture was then heated at 50 °C for 30 minutes. Finally, RNAase inactivation carried out for five minutes at 95 °C.

###  Real time quantitative PCR

 Real-time PCR amplification and detection were carried out utilizing the quantitative iQ5 real-time PCR detection equipment (Bio-Rad Laboratories Inc., Hercules, USA) in order to assess the expression levels of certain genes linked to apoptosis. Reactions made with the BioFact^TM^ real-time PCR master mix was used for this. As indicated in Table 1, primers were designed using Oligo 7.56 (Molecular Biology Insights, Inc., USA) via public Gene Bank sequences. A total volume of 25 µl was used for all amplification reactions. The following ingredients were used in each well: 1 µl cDNA, 1 µl primer (100 nM for each primer), 12.5 µl 2X SYBR Green PCR Master Mix, and 10.5 µl RNAse/DNAse free water. The following thermal cycling parameters were used: one cycle at 94 °C for 10 min, 40 cycles at 95 °C for 15 seconds, 30 seconds at 59–61 °C (depending on the gene), and 25 seconds at 72 °C. Cycle threshold (CT) data were adjusted to the level of ß-actin expression as a housekeeping gene. Each experiment contained a negative control, no template control as well as triple runs of each reaction.

**Table 1:** Primers sequences

**Primer**	**Sequence**	**Tm (°C)**	**Running conditions**
*β -actin*	F: 5’-TGCCCATCTACGAGGGGTATG-3’ R: 5’-CTCCTTAATGTCACGCACGATTTC-3’	59	- One cycle at 94 °C for 10 min, - 40 cycles at 95 °C for 15 seconds, - 30 seconds at Tm, - 25 seconds at 72 °C.
*Casp3*	F: 5’-TGCCTGTAACTTGAGAGTAGATGG-3’ R: 5’-CTTCACTTTCTTACTTGGCGATGG-3’	61	
*Bcl2*	F: 5’-CATCAGGAAGGCTAGAGTTACC-3’ R: 5’-CAGACATTCGGAGACCACAC-3’	60	
*BCL-XL*	F: 5’- GTTCCCTTTCCTTCCATCC -3’ R: 5’- TAGCCAGTCCAGAGGTGAG -3’	59	
*NOX4 *	F: 5’-GCTGCATCAGTCTTAACCGAAC-3’ R: 5’- GGCTCTTCCATACAAATCTTCACA-3’	60	

###  Statistical analysis

 The statistical studies were performed using one-way analysis of variance (ANOVA) followed by *post-hoc *Tukey multiple comparison test (SPSS 13.0 software; SPSS Inc., Chicago, IL, USA). Results were all displayed as mean ± SD. Statistical significance was defined as a *P* value < 0.05.

## Result

###  Free radical scavenging assessment

**Figure 2:**
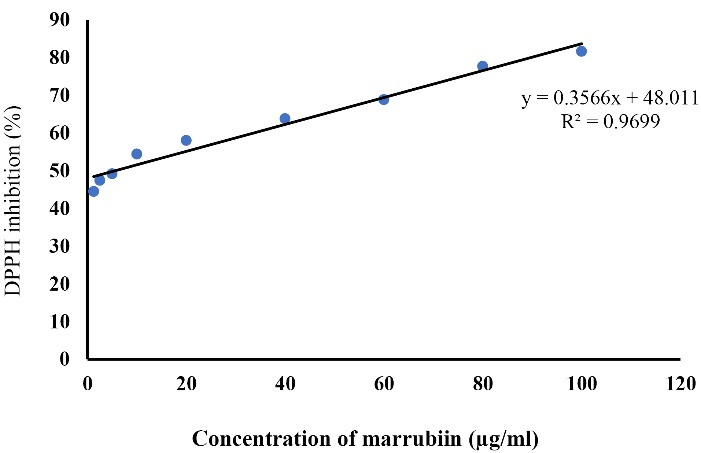
DPPH free radical scavenging assay for detection of EC_50_ of marrubiin. DPPH: 2, 2-diphenyl-1-picrylhydrazyl, EC50: half maximal effective concentration

 The Antioxidant property of marrubiin was evaluated using the DPPH assay, which is commonly used to assess the free radical scavenging capacity of natural products based on measuring the reduction of free radicals. Our results showed that marrubiin is able to inhibit the free radical scavenging activity with the EC50 value of 16.7 µM (Figure 2).

###  Marrubiin effects on intracellular GSH level

**Figure 3:**
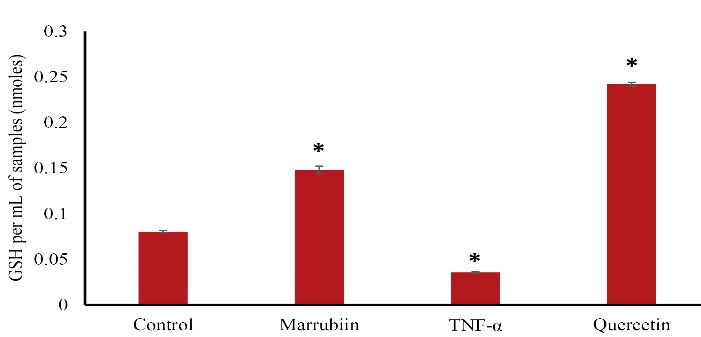
Effects of marrubiin on GSH levels in TNF-α induced HUVECs. The GSH level significantly decreased in TNF-α treated HUVECs. Whereas marrubiin have increased the GSH level significantly in TNF-α induced HUVECs (*p* < 0.05). The asterisk represents significant differences between treatment conditions with untreated cells (*p* < 0.05). GSH: glutathione, HUVECs: Human umbilical vein endothelial cells, TNF-α: Tumor necrosis factor alpha

 To assess Marrubiin’s potential in protecting endothelial cells from damage caused by TNF-α, we examined intracellular levels of GSH in this investigation. The findings demonstrated that TNF-α- treated HUVECs significantly reduced the intracellular amount of GSH in (Figure 3).

###  Marrubiin effects on TNF-α induced apoptosis/oxidative stress genes levels 

**Figure 4:**
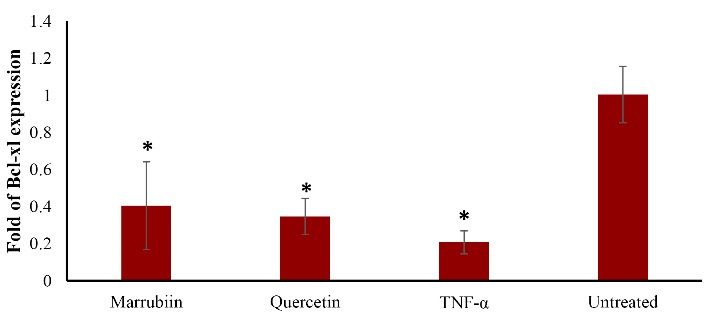
Effect of marrubiin on Bcl-xl expression in TNF- α treated HUVECs. TNF- α (50 ng/ml) attenuates Bcl-xl gene expression level however marrubiin could significantly reverse this effect and increase Bcl-xl gene expression level in HUVECs. The asterisk represents significant differences between treatment conditions with untreated cells (*p* < 0.05). TNF-α: Tumor necrosis factor alpha, HUVECs: Human umbilical vein endothelial cells, Bcl-xl: B-cell lymphoma-extra large

 Using quantitative real-time PCR (qPCR), we evaluated the effects of marrubiin on TNF-α-induced oxidative stress and apoptotic gene levels. Compared to TNF-α-treated cells, the expression level of Bcl-xl considerably increased in marrubiin-treated cells (Figure 4) (*p* < 0.05). Although the amount of Bcl-xl expression was higer than that of TNF-α-treated cells, it was not statistically significant. The experiment’s findings demonstrated that marrubiin dramatically increased the expression of the antiapoptotic gene Bcl-xl in cells treated with TNF-α.

**Figure 5:**
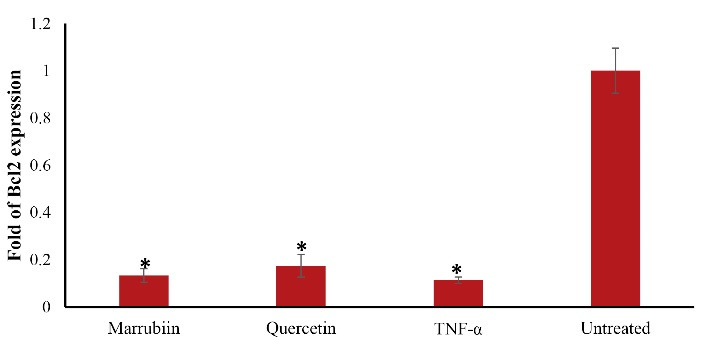
Effect of marrubiin on Bcl2 expression in TNF- α treated HUVECs. TNF- α (50 ng/ml) attenuates Bcl2 gene expression level however marrubiin could increase Bcl2 gene expression level in HUVECs but it was not significant. The asterisk represents significant differences between treatment conditions with untreated cells (*p* < 0.05). TNF-α: Tumor necrosis factor alpha, HUVECs: Human umbilical vein endothelial cells, Bcl2: B-cell lymphoma 2

 The expression level of Bcl2 also increased in marrubiin-treated cells compared to TNF-α-treated cells, although the difference was not statistically significant (*p* > 0.05) (Figure 5). Although Bcl-2 expression was higher than that of TNF-α-treated cells, this difference was also not statistically significant.

**Figure 6:**
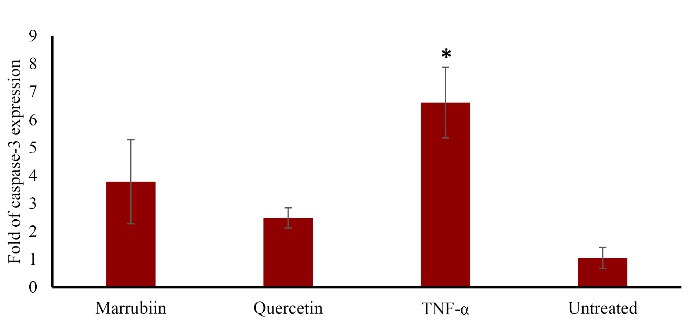
Effect of marrubiin on caspase-3 expression in TNF- α treated HUVECs. TNF- α (50 ng/ml) enhances caspase-3 gene expression level however marrubiin could significantly reverse this effect and attenuates caspase-3 gene expression level in HUVECs. The asterisk represents significant differences between treatment conditions with untreated cells (*p* < 0.05). TNF-α: Tumor necrosis factor alpha, HUVECs: Human umbilical vein endothelial cells

 TNF-α increases the expression of the caspase-3 gene in HUVECs. Our findings demonstrated that marrubiin significantly reduced the expression of caspase-3 in TNF-α-treated HUVECs (*p* < 0.05) (Figure 6).

**Figure 7:**
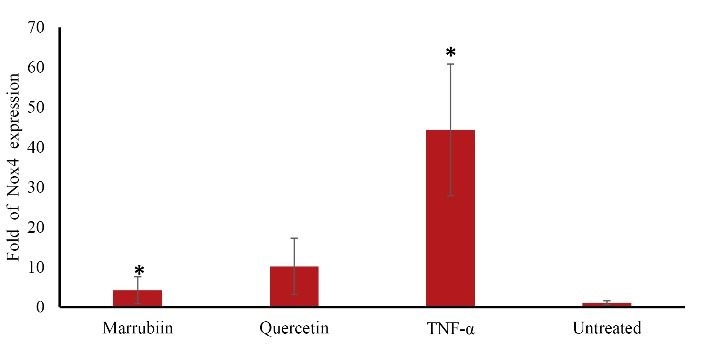
Effect of marrubiin and on Nox4 expression in TNF- α treated HUVECs. TNF- α (50 ng/ml) enhances Nox4 mRNA expression level however marrubiin could significantly reverse this effect and attenuates Nox4 gene expression level in HUVECs. The asterisk represents significant differences between treatment conditions with untreated cells (*p* < 0.05). TNF-α: Tumor necrosis factor alpha, HUVECs: Human umbilical vein endothelial cells, NOX4: NADPH Oxidase 4

 Our findings indicated that Nox4 is overexpressed in TNF-α-treated cells, but treatment with marrubiin dramatically reduced Nox4 mRNA expression significantly. (*p* < 0.05) (Figure 7).

## Discussion

 Atherosclerosis is a chronic inflammatory disease characterized by the accumulation of lipid deposits, inflammatory cells, and fibrous tissue in the arterial walls. Oxidative stress plays a crucial role in the development and progression of atherosclerosis. Marrubiin a plant-derived compound, possesses promising therapeutic potential. Marrubiin’s ability to scavenge ROS and inhibit lipid peroxidation makes it an attractive candidate for preventing atherosclerosis.

 It is speculated that marrubiin is a potential antioxidant. These results are in line with reliable studies demonstrating marrubiin antioxidant capacity.^12,13^

 It has long been known that there is a link between glutathione metabolism and cardiovascular disease, and experimental studies have added to this evidence.^14^ The GSH redox cycle is the most significant antioxidant mechanism in endothelial cells, and it has been demonstrated that many cardiovascular disorders, including atherosclerosis are accompanied by GSH depletion.^15^ Additionally, it is well known that in hyperlipidemic mice, pharmacological treatment and an increase in GSH levels might somewhat change the onset of atherosclerotic lesions. It was shown that enhancing macrophages’ ability to produce GSH offers some protection against lesion development even when lesions have already formed. Furthermore, it is well known that intracellular GSH is essential for endothelial cells’ defense against oxidized Ox-LDL and that the loss of intracellular GSH contributes to OX-LDL toxicity.^16^ By increasing NO activity, thiol supplementation with GSH specifically alleviates human endothelium dysfunction.^14^

 High GSH concentrations may give resistance to oxidative stress-induced apoptosis because cellular redox status may control cell viability. Our findings demonstrated that marrubiin has the ability to partially counteract TNF-α’s effects on the levels of GSH in HUVECs. The findings are consistent with our earlier research, which demonstrated that marrubiin has the ability to inhibit free radical scavenging activity with an IC50 value of 16.7 µM. The results indicate that marrubiin has the potential to significantly reduce TNF-α-induced intracellular ROS generation in HUVECs.^12^ Additionally, these results are in agreement with other studies that suggested marrubiin has cardioprotective and antioxidant charactristics.^17-19^ Given that oxidative stress caused by TNF-α has been proven to play a significant role in CVDs, this may also suggest the possible utility of marrubiin in such diseases. Moreover apoptosis is involved in various stages of atherosclerosis, including endothelial cell dysfunction, foam cell formation, and plaque destabilization. Excessive apoptosis of endothelial cells and vascular smooth muscle cells contributes to plaque vulnerability and rupture. Inhibiting apoptosis may maintain the stability of atherosclerotic plaques and reduce the risk of cardiovascular events.

 The increase in Bcl-xl gene expression showed that marrubiin has the ability to prevent endothelial cells from apoptosis and the onset and development of atherosclerosis. The negligible increase of Bcl2 could be linked to the duration of the treatments. Therefore, a longer period of time of marrubiin treatment would be needed to affect Bcl-2 gene expression. Atherosclerotic plaques produce the apoptosis protease caspase-3, which is linked to apoptotic cells that cause plaque rupture.^20^ Apoptosis not only causes the atherosclerotic plaque to become fragile and rupture, but it also stimulates the absorption of OX-LDL by the endothelium by turning macrophages into foam cells.^21^ Therefore, suppressing the genes that trigger apoptosis may be a successful strategy to prevent atherosclerosis from progressing. In human atherosclerosis, NOX is increased and primarily contributes to the generation of ROS, according to experimental and clinical findings. The NOX family is composed of NOX1, NOX2, NOX3, NOX4, and NOX5. The most significant NOX isoforms in vascular cells are NOX1, NOX2, and NOX4. Numerous investigations have shown that NOX activation may be involved in the development and progression of atherosclerosis.^22^ Nox4 differs from other isoforms due to the high level of expression it, exhibits in cardiovascular tissue. All vascular cells, endothelial cells, and VSMCs have strong Nox4 expression, which suggests that it is always active. One of the primary mechanisms by which cells produce ROS is NADPH oxidase. Furthermore, Nox4-NADPH oxidase controls adipogenesis, which is a risk factor for atherosclerosis.^23^ This is mediated by insulin-induced pre-adipocyte differentiation, and cytosolic regulatory factors such as polymerase delta-interacting protein 2 activate Nox4 and control VSMC migration. A great deal of attention has been paid to ROS-generating pathways that have been implicated in ROS overexpression in endothelial dysfunction and vascular inflammation, both of which are major factors in human atherosclerosis. NADPH oxidase is the most significant enzyme responsible for ROS generation in human arteries among the enzymatic pathways. This enzyme has been implicated in the onset and development of atherosclerotic disease which approved in both experimental and clinical trials.^24^

 The combined antioxidant and anti-apoptotic properties of marrubiin make it a promising candidate for preventing atherosclerosis. Its ability to scavenge ROS and inhibit lipid peroxidation can protect endothelial cells from oxidative damage and preserve their function. Additionally, by suppressing apoptosis, marrubiin may contribute to the stability of atherosclerotic plaques, reducing the risk of plaque rupture and subsequent thrombotic events.

## Conclusion

 It has been established that NOX4/ROS, which is primarily triggered by TNF-α, plays a significant role in the development of atherosclerosis. To have the greatest impact on coronary heart disease, antioxidant treatment should be thought of as an adjuvant to lipid-lowering treatment approach. Public health initiatives and medical intervention might both be used to prevent oxidation-induced atherosclerosis. The quantification of intracellular ROS in the current study verified the protective effect of marrubiin against TNF-induced intracellular oxidative stress. Cardiocytotoxicity was demonstrated to be closely connected with the amount of TNF-α-induced mitochondrial ROS, and the ROS generation could be efficiently scavenged by the mitochondrial glutathione system. Here, marrubiin demonstrated excellent potential for maintaining cell GSH level. Along with increasing Bcl-xl mRNA expression, marrubiin inhibited TNF-α-induced caspase-3 and Nox4 mRNA overexpression. It is well known that GSH levels affect Bcl-2 expression and function, transcription factor activation, caspase activity, and the effects of marrubiin. Previously, we established the free radical shielding characteristics of marrubiin utilizing a variety of tests, including DNA fragmentation, cell cycle, and apoptosis detection. NADPH oxidase-mediated and mitochondrial-mediated ROS production are thought to be the two primary causes of CVDs, and marrubiin may control both pathways. In this study, pretreatment with the antioxidant reduced TNF-α-induced cell damage, partially due to antiapoptotic actions on endothelial cells. Furthermore, the findings suggested that marrubiin might be a powerful protective entity in diseases associated with elevated levels of intracellular ROS. Given the importance of intracellular ROS in the development and progression of different diseases (e.g., neurological or cardiovascular diseases, and cancer), we suggest marrubiin as an effective adjuvant treatment to regulate such disorders. To support such an application, in vivo investigations in CVD model animals should be carried out. Furthermore, more research is needed to understand the underlying processes and pathways of marrubiin’s protective action.

## Acknowledgments

 Thanks for Professor Nazemiyeh, Tabriz University of Medical Sciences, and Tabriz, Iran, who provided Marrubiin as a courtesy gift.

## Competing Interests

 The authors declare no conflicts of interest concerning this article.

## Ethical Approval

 Not applicable. This paper does not involve research on animals or humans.

## Funding

 This work is a part of a Ph.D thesis supported (grant No: RCPN-93014) by Research Center for Pharmaceutical Nanotechnology, Biomedicine Institute, Tabriz University of Medical Sciences.
